# Prevalence of Deliberate Self-harm Among Chinese Patients With Heroin Dependence: A Meta-Analysis

**DOI:** 10.3389/fpsyt.2018.00325

**Published:** 2018-07-18

**Authors:** Bao-Liang Zhong, Yan-Min Xu, Wu-Xiang Xie, Wen-Cai Chen, Jin Lu

**Affiliations:** ^1^Research Center for Psychological and Health Sciences, China University of Geosciences, Wuhan, China; ^2^Affiliated Wuhan Mental Health Center, Tongji Medical College, Huazhong University of Science and Technology, Wuhan, China; ^3^Peking University Clinical Research Institute, Peking University Health Science Center, Beijing, China; ^4^Department of Psychiatry, The First Affiliated Hospital of Kunming Medical University, Kunming, China

**Keywords:** deliberate self-harm, prevalence, heroin dependence, detoxification, meta-analysis

## Abstract

**Background:** There is paucity of data regarding the prevalence and methods of deliberate self-harm (DSH) in patients with heroin dependence in international literature. In China, there have been a few studies investigating the prevalence of DSH in heroin-dependent patients (HDPs), but their rates varied widely. We thus conducted a meta-analysis of studies assessing the prevalence of DSH among Chinese HDPs.

**Methods:** Relevant studies were retrieved from major Chinese databases (China National Knowledge Infrastructure, Wanfang data, and SinoMed) and western databases (PubMed, EMBASE, and PsycInfo). Two authors independently identified eligible studies and extracted data. Studies that included a representative sample of Chinese HDPs and ascertained DSH caseness in a reliable way were considered as high quality. Statistical analysis was performed using R software.

**Results:** In total, 15 eligible studies with a total of 37,243 Chinese HDPs were included. All included studies were conducted in heroin detoxification settings. Only two studies were rated as high quality. The pooled prevalence of DSH in Chinese HDPs was 4.4% (95%CI: 2.9, 6.2%), but the heterogeneity of prevalence rates across studies was significant (*I*^2^ = 98%, *P* < 0.001). Studies rated as high quality had significantly higher prevalence of DSH than those rated as low quality (13.2 vs. 3.4%, *P* < 0.001). Swallowing foreign objects was the most common method of DSH, with a combined prevalence of 2.7% (95%CI: 1.6, 4.4%). Extreme DSH methods such as cutting off fingers and jumping from height were also not uncommon in this patient population.

**Conclusion:** Due to methodological problems in available studies, we find a relatively low prevalence of DSH among Chinese HDPs receiving detoxification treatment. Nevertheless, the self-harmers of Chinese HDPs are more likely to harm themselves in a dangerous or life-threatening way. Restricting the availability of DSH methods may be an effective way to prevent or reduce DSH in China's detoxification treatment settings.

## Introduction

Deliberate self-harm (DSH) is a health risk behavior defined as an act of intentional bodily harm without apparent suicidal intent (1). DSH is one of the most common self-destructive behaviors in patients with substance use disorders such as alcohol dependence, cannabis abuse, and other illicit drug use (2–5). In addition to injuries, infections, and tissue damage resulted from DSH, studies have demonstrated that DSH is significantly associated with a variety of psychosocial problems such as depression, anxiety, and loneliness, functional impairment, poor quality of life, premature death, later attempted suicide, and subsequent suicide (6–10). Further, DSH also has major negative impacts on the self-harmer's loved ones, including psychological distress, feelings of guilt, social isolation, and stigma (11). Therefore, the identification and treatment of DSH is an essential component of clinical management of patients with drug use disorders.

Worldwide, opioids are the most harmful drug, representing 70% of the burden of disease attributable to drug use disorders (12), and heroin is the second most common drug of abuse in China (13). Therefore, clinical management of heroin (and other opioids) dependence is one of the highest priorities of psychiatric services for many countries in the world, including China. In contemporary China, compulsory detoxification is the main type of treatment for illicit drug dependence, managed by departments of public security of the government. This is supplemented by voluntary detoxification services, provided by psychiatric specialty hospitals (14). In 2016, a total of 661,000 newly-registered illegal drug users were receiving treatment in mainland China: 91.1% compulsory and 8.9% voluntary (15). However, due to healthcare providers' limited knowledge and skills in managing self-harm, DSH might be an underecognized and undertreated issue in drug dependence treatment settings (14, 16, 17); this situation is particularly serious in China's compulsory detoxification settings because of a shortage of medical professionals in these institutions (14).

DSH is also prevalent in the general population, in particular younger age-groups, and it manifests in various forms, with cutting accounting for approximately 70% of the individuals who harm themselves (18, 19). Because heroin acts upon the central nervous system and results in alterations in the users' perception, mood, consciousness, attention or behavior, rates and methods of DSH of heroin-dependent patients (HDPs) might be different from those of the general population. Nevertheless, in international literature, there is a dearth of studies examining DSH in HDPs. To the best of our knowledge, only one study reported a lifetime DSH prevalence of 25% in a sample of opioid-dependent patients in Australia but the clinical characteristics of self-harmers such as DSH methods have not been fully elucidated (20). By contrast, there have been some studies on the DSH of HDPs in China (21–35). However, most of these available studies were published in Chinese-language journals, and are therefore less accessible to the international readership.

DSH patients with heroin dependence present a considerable challenge for clinical services. A greater understanding on the prevalence and methods of DSH would help address this important clinical problem. Existing Chinese studies have reported a wide variations in the prevalence of DSH among HDPs (1.1–17.7%) and presented a mixed profile of DSH methods (21, 26, 32). To help clarify these issues, this meta-analysis quantitatively synthesized the results of relevant studies to determine the prevalence rates of DSH and its methods of Chinese HDPs.

## Materials and methods

This meta-analysis is reported according to the Preferred Reporting Items for Systematic Reviews and Meta-Analyses and Meta-analysis of Observational Studies in Epidemiology guidelines.

### Search strategy

We searched potential articles with search engines including PubMed, Embase, PsycInfo, China National Knowledge Infrastructure, Wanfang, and SinoMed. To avoid omission of relevant studies, we also hand-searched the reference lists of all identified relevant papers and reviews. Keywords used for the literature search included (heroin OR opiate OR opioids OR abstinence OR narcotic OR detoxification) AND (self-harm OR self-injuri^*^ OR self-mutilation OR parasuicide OR self-destruct^*^ OR self-inflict^*^ OR self-wound^*^). All papers published by March 20, 2018 were included.

### Selection criteria

Inclusion criteria were (1) cross-sectional studies with meta-analyzable data (i.e., reporting the DSH prevalence or number of patients with DSH and sample size); (2) study subjects were Chinese HDPs, regardless of other illicit drugs of abuse (as long as heroin was the main drug of abuse); and (3) the presence of DSH could be determined by any approaches (i.e., clinical interview, self-report questionnaire) but the act of self-harm should be done without suicidal intent.

### Data extraction

The following information were independently extracted from each included study: first author, publication year, study period, sample characteristics (percentage of males and mean age), diagnostic criteria of heroin dependence, type of treatment, assessment of DSH, sampling method, response rate, sample size, number of DSH patients, and numbers of patients with different subtypes of DSH.

### Quality assessment

The validity of prevalence studies that involve psychiatric patients is mainly determined by the sample representativeness and the accuracy of outcome assessment (14, 36, 37). In this study, the former depends on sample size, sampling method, and response rate, and the latter is determined by the validity of the assessment (14, 37). In the case of our study, studies having a minimum number of 100 HDPs, recruiting subjects by using cluster sampling, having a response rate of 70% and above, and assessing DSH in a reliable way (i.e., with validated standardized scales such as NSSI-AT [Non-Suicidal Self-Injury-Assessment Tool] and clinical interview) were regarded as having high quality, whereas studies having a sample of 99 HDPs or fewer, recruiting subjects by using convenient sampling, having a response rate of less than 70% or assessing DSH in a questionable way (i.e., with unvalidated or unstandardized scales) were regarded as having poor quality (14).

Two authors (YMX and WCC) independently searched literature databases, evaluated the eligibility of potential studies, extracted data, and assessed quality of included studies. Any discrepancies were resolved by consensus of the two authors after reviewing the primary data again.

### Statistical analysis

We used meta-analysis to produce combined estimates and their 95% confidence intervals (CIs) for the prevalence rates of DSH and its methods in the whole sample. Forest plots were used to show the prevalence rates and the combined estimates. When there was evidence of heterogeneity (*I*^2^ > 50%), prevalence rates were combined by using a random-effect model; otherwise, rates were combined by using the fixed-effect model. Sources of heterogeneity in the prevalence estimates of DSH were explored by subgroup analyses according to sample characteristics (reported vs. not reported), diagnostic criteria for heroin dependence (reported vs. not reported), middle year of the observation period (<2,000 vs. ≥2,000), type of detoxification (compulsory vs. voluntary), study quality (high vs. low), and sample size (≤1,255 vs. >1,255). Significance of differences in DSH prevalence estimates between subgroups was tested with Q-value test. We used funnel plot and Begg's/Egger's tests to test publication bias. Rates were transformed using the Freeman-Turkey variant of the arcsine square root transformation before pooling. All analyses were performed using R 3.1.1 (R Development Core Team; Vienna, Austria).

## Results

**Figure 1:**
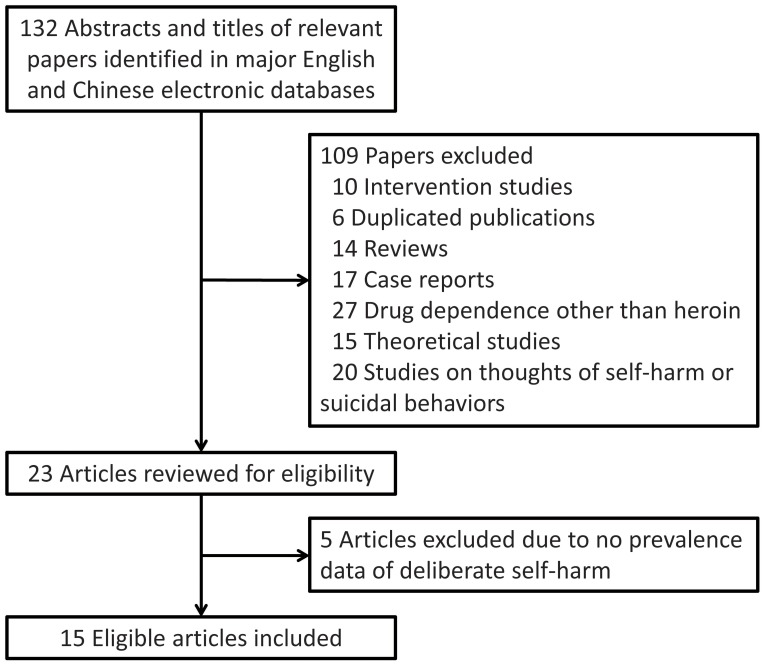
Flowchart of study inclusion.

Our literature search identified 132 unique candidate articles, of which 117 were excluded due to different reasons (Figure 1). Finally, 15 studies with a total of 37,243 Chinese HDPs were included in this meta-analysis (21–35). All included studies were published in Chinese. HDPs of 14 studies were receiving compulsory detoxification treatment and patients of the remaining one were receiving voluntary detoxification treatment (31). Therefore, subjects of this meta-analysis were limited to HDPs of detoxification institutions. In total, these included studies reported over ten methods of DSH. Characteristics of the included studies were displayed in Table 1.

**Table 1:** Basic characteristics, quality, and numbers of DSH patients by methods of included studies.

**Study**	**Sample characteristics (% men, % of age-group or mean age)**	**Diagnostic criteria for heroin dependence**	**Observation period**	**Type of treatment**	**DSH assessment**	**Sampling**	**Response rate (%)**	**Quality**	**No. of self-harmers**	**No. of HDPs**	**No. of HDPs by DSH methods**
Shen, et al. (22)	NR	DSM-III-R	1990–96	C	MR	C	100	Low	200	14,000	S:132, C:25, B:24, J:14, D:3, O:2
Zhu, et al. (23)	75.3% men, 30.3 years	NR	1996–98	C	MR	C	100	Low	32	591	S:21, C:9, H:2
Luo, et al. (24)	74.3% men, 90.3% <35 years	NR	1994–98	C	MR	C	100	Low	51	1,255	S:37, C:12, H:2
Feng, et al. (35)	NR	NR	2001–02	C	MR	C	100	Low	80	4,523	S:66, C:9, B:1, H:2, O:2
Wang, (26)	NR	NR	1998–99	C	CI & PE	C	100	High	58	327	S:45, C:10, H:3
Liu, (27)	NR	NR	1999–2000	C	MR	C	100	Low	95	883	S:21, C:8, B:55, H:10, Cu:1
Yang, (28)	NR	NR	1998–2003	C	MR	C	100	Low	8	627	NR
Xia, et al. (21)	NR	CCMD-3	2001–02	C	MR	C	100	Low	20	1,760	NR
Jia, (29)	NR	NR	1996–2002	C	MR	C	100	Low	42	527	S:42
Lu, et al. (30)	NR	NR	1997–2002	C	MR	C	100	Low	102	2,108	S:35, C:65, Br:1, K:1
Tao, et al. (31)	NR	DSM-IV	2002–03	V	MR	C	100	Low	8	196	NR
Zhang, et al. (32)	85.3% men, 31 years	DSM-III-R	1997–2001	C	CI & PE	C	100	High	315	3,258	S:185, C:96, B:13, Cu:2, J:4, A:8, O:7
Wang, (33)	NR	NR	2001–03	C	MR	C	100	Low	52	2,827	S:44, C:4, A:1, O:3
Wu, et al. (34)	NR	DSM-IV	2002–06	C	MR	C	100	Low	79	3,841	S:61, O:18
Luo, (35)	NR	DSM-IV-R	2008–09	C	MR	C	100	Low	20	520	S:6, H:14

*DSH, deliberate self-harm; NR, not reported; HDPs, heroin-dependent patients*.

*Sample characteristics: percentage of males and mean age (or percentage of an age-group)*.

*Diagnostic criteria for heroin dependence: DSM, Diagnostic and Statistical Manual of Mental Disorders; CCMD, Chinese Classification and Diagnostic Criteria of Mental Disorders*.

*Type of treatment: C, compulsory detoxification; V, voluntary detoxification*.

*DSH assessment: MR, medical records; CI and PE, clinical interview and physical examination*.

*Sampling: C, cluster sampling*.

*DSH methods of self-harm: S, swallowing foreign objects such as broken glass, iron nails, keys, lighter, paperclip, scissors, belt fastener, and nail clipper; C, cutting oneself with broken glasses, chopsticks, ring-pull can, and so on; B, burning oneself with cigarette, hot water, and so on; H, hitting oneself with a hard object and hands, or hitting heavy objects (such as a wall); Cu, cutting off fingers; J, jumping from height; Br, breaking bones; K, knocking a nail into the head; A, apastia; D, drug overdose; O, others*.

Two studies assessed the presence of DSH by using a combined approach of clinical interview and physical examination (26, 32), and the rest assessed DSH based on a retrospective review of medical records. Although DSH cases ascertained by the two approaches excluded self-harmers with suicidal intent, we considered that the later assessment was questionable, because it is very likely that only moderate-to-severe DSH cases that need medical treatment could be identified in the medical records. All studies chose subjects by using cluster sampling (i.e., non-selectively included all HDPs of an institution during the observation period), and therefore the response rates were all 100%. Sample sizes of included studies ranged between 196 and 14,000, with a median value of 1,255. As a results of these reasons, only two studies were rated “high” for the overall quality rating, and the remaining 13 studies were rated “low” (Table 1).

**Figure 2:**
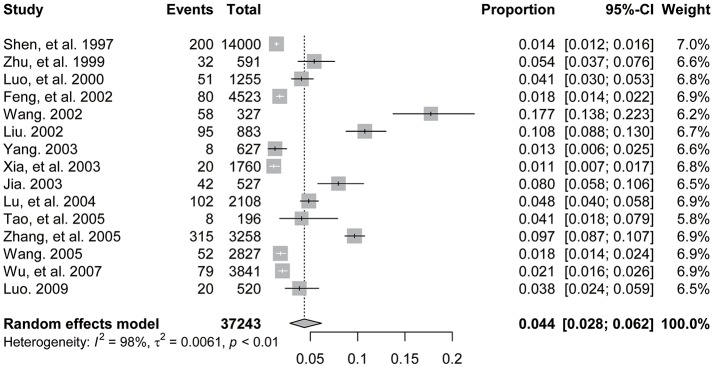
Forest plot of prevalence of deliberate self-harm among Chinese patients with heroin dependence.

A high level of heterogeneity was found in the prevalence of DSH across all included studies (*I*^2^ = 98%, *P* < 0.001). The pooled prevalence (95%CI) of DSH from the random-effect model among Chinese HDPs was 4.4% (2.8, 6.2%) (Figure 2). The combined rates of DSH methods of Chinese HDPs (in order of prevalence) were 2.7% (95%CI: 1.6, 4.4%) for swallowing foreign objects, 0.9% (95%CI: 0.4, 1.9%) for cutting, 0.5% (95%CI: 0.2, 1.5%) for hitting, 0.4% (95%CI: 0.01, 3.3%) for burning, 0.1% (95%CI: 0.03, 0.2%) for jumping from height, 0.1% (95%CI: 0.01, 0.2%) for cutting off fingers, 0.1% (95%CI: 0.01, 0.7%) for apastia, 0.05% (95%CI: 0.001, 0.3%) for breaking bones, 0.05% (95%CI: 0.001, 0.3%) for knocking a nail into the head, and 0.02% (95%CI: 0.001, 0.1%) for drug overdose.

**Figure 3:**
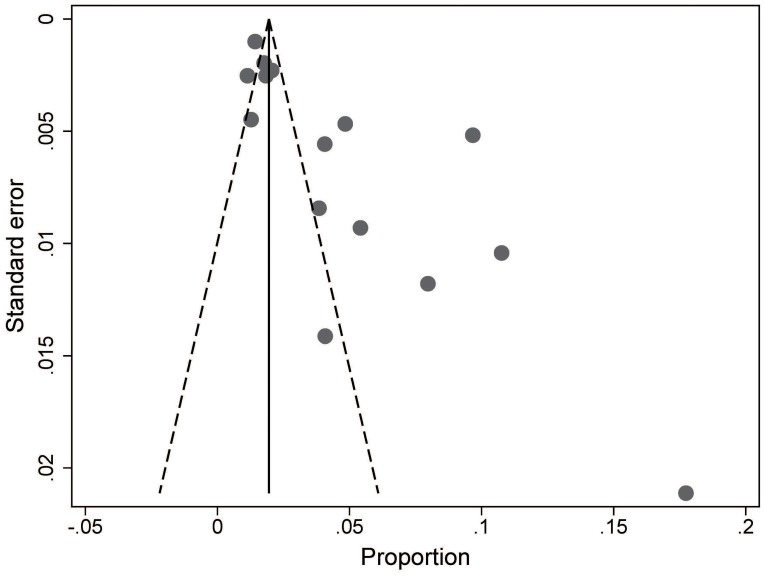
Funnel plot of publication bias among the 15 included studies.

Although the shape of the funnel plot seems asymmetrical (Figure 3), results of Egger's test (*t* = 0.529, *P* = 0.606) and Begg's test (*Z* = 0.148, *P* = 0.882) showed that there was no significant publication bias in all included studies.

Results of subgroup analyses are displayed in Table 2. The prevalence of DSH was significantly higher in studies conducted before 2000 (vs. those conducted after 2000) and in studies rated as high quality (vs. those rated as low quality).

**Table 2:** Subgroup analysis of the source of heterogeneity of included studies.

**Factor**	**No. of studies**	**No. of DSH cases**	**No. of HDPs**	**Heterogeneity (*I*^2^, *P*)**	**Pooled rate (%, 95%CI)**	**Q**	**P**
**SAMPLE CHARACTERISTICS**							
Not reported	12	764	32,139	97%, *P* < 0.001	3.9 (2.6, 5.5)		
Reported	3	398	5,104	96%, *P* < 0.001	6.2 (3.0, 10.6)	1.995	0.158
**DIAGNOSTIC CRITERIA FOR HEROIN DEPENDENCE**							
Not reported	9	520	13,668	97%, *P* < 0.001	5.2 (3.1, 7.9)		
Reported	6	642	23,575	99%, *P* < 0.001	3.2 (1.2, 6.2)	0.775	0.379
**MIDDLE YEAR OF THE OBSERVATION PERIOD**							
<2000	8	895	22,949	99%, *P* < 0.001	7.0 (3.6, 11.4)		
≥2000	7	267	14,294	70%, *P* < 0.001	1.9 (1.4, 2.4)	10.309	0.001
**TYPE OF DETOXIFICATION**							
Compulsory	14	1,154	37,047	98%, *P* < 0.001	4.4 (2.8, 6.3)		
Voluntary	1	8	196	–	4.1 (1.7, 7.4)	0.032	0.858
**SAMPLE SIZE**							
≤1,255	8	314	4,926	95%, *P* < 0.001	6.2 (3.5, 9.5)		
>1,255	7	848	32,317	99%, *P* < 0.001	2.8 (1.3, 4.8)	3.551	0.06
**STUDY QUALITY**							
High	2	373	3,585	94%, *P* < 0.001	13.2 (6.4, 22.1)		
Low	13	789	33,658	96%, *P* < 0.001	3.4 (2.3, 4.6)	13.757	<0.001

## Discussion

This meta-analysis summarized studies reporting the prevalence rates of DSH and its methods among Chinese HDPs receiving detoxification treatment. We found that 4.4% Chinese HDPs had comorbid DSH and that the most common DSH method was swallowing foreign objects (2.7%), accounting for 61.4% of the self-harmers. Further, some other extreme forms of DSH such as jumping from height (0.1%), cutting off fingers (0.1%), and breaking bones (0.05%) were also not uncommon in Chinese HDPs.

In the literature, DSH prevalence rates in adolescents (7.5–46.5%) and adults (4–23%) vary considerably (38). Many factors may contribute to variation in DSH rates, including differences in definition (i.e., self-harming behavior vs. non-suicidal self-injury disorder), assessment (i.e., clinical interview vs. self-report questionnaire), and timeframe of interest (i.e., lifetime vs. twelve-month), and developmental context. With regard to DSH among international patients with substance use disorders, a 1-week prevalence of 6.9% and a lifetime prevalence of 25.0–45.5% have been reported (20, 39–41). It seems that the risk of DSH in Chinese HDPs receiving detoxification treatment is lower than that in the general population and international patients with substance use disorders. However, this may not be true because the current study focused on the prevalence of DSH during the period of detoxification treatment only, not DSH in the past week/year or during the lifetime. The other explanation for the relatively low pooled prevalence of DSH found in this meta-analysis is the problematic assessment of DSH used in most of the included studies—based on medical records only, an approach would miss quite a lot of cases with minor DSH that requires no medical attention. This speculation is supported by the results of subgroup analysis based on study quality: the pooled DSH prevalence of studies based on clinical interview and physical examination is nearly 4-fold higher than that of studies based on medical records (13.2 vs. 3.4%). In addition, one of our included studies compared the prevalence of DSH between HDPs under compulsory detoxification and prisoners of theft and found a significantly higher risk of DSH in HDPs than prisoners (1.28 vs. 0.27%) (28). Due to these reasons, the true prevalence of DSH in Chinese HDPs receiving treatment should be higher than what we observed in this study, at least 13.2%.

DSH generally occurs when someone intentionally herself/himself in a way that is not intended to be lethal. In adolescents and adult population, the most frequent method of DSH is self-cutting, followed by head banging scratching, hitting, and burning (38). In a mixed sample of patients with substance (cannabis, heroin, phensedyl, alcohol, and other drugs) use disorders, the two most common types of DSH were cutting and burning, representing 39.0 and 26.0% of the self-harmers, respectively (41). On the contrary, the meta-analysis found a different pattern of DSH methods in Chinese HDPs: swallowing foreign objects is the most common, and some other more lethal forms of DSH such as jumping from a high place and knocking a nail into the head occur too. A prognostic analysis of 48 Chinese HDPs who intentionally swallowed sharp foreign objects reported that 38 (79.2%) patients received surgical treatment and one patient (2.1%) died due to hemorrhagic shock (42). Therefore, our data suggest that the self-harmers of Chinese HDPs have a higher likelihood of harming themselves in a dangerous or life-threatening way.

Until now, empirical studies have provided evidence for some functions of DSH, including affect-regulation and self-punishment (43, 44). Because DSH is a way to quickly relieve overwhelming negative feelings, the severe painful feelings and depressive and anxiety symptoms of the HDPs may be the primary reason for DSH during the acute withdrawal period. On the other side, HDPs may feel guilt for their past misconduct behaviors and punish themselves for being addicts. Intolerable withdrawal symptoms, feelings of guilt and emptiness, public stigma toward persons with drug addiction, being abandoned by family members, and even death wish may together result in the more lethal DSH methods of HDPs. In China, compulsory detoxification treatment is also an administrative punishment for HDPs who refused voluntary detoxification or relapse on heroin. Therefore, HDPs may also use self-harm, in particular more fatal methods of self-harm, as a means to escape punishment, i.e., getting the chance of being released on bail for medical services (24).

Subgroup analyses found that in addition to difference by study quality, the study's observation period was also associated with the pooled prevalence of DSH among Chinese HDPs. The low DSH prevalence in 2000 and onwards relative to before 2000 might be a result of the improvement in the clinical management of heroin dependence in recent years.

Our study has several limitations. First, 13 of the 15 included studies were of low quality, and our subgroup analysis found significantly lower DSH prevalence in low quality studies. Thus, as we discussed above, this meta-analysis under-estimates the true prevalence. Second, there was significant heterogeneity in the estimated DSH prevalence across studies, even after the subgroup analyses according to some potential sources of heterogeneity. Third, due to incomplete reporting of the results of included studies, the gender difference in DSH prevalence could not be examined in this study. Finally, subjects of all included studies were those receiving detoxification treatment at institutions. We did not identify any studies on DSH of untreated patients, patients of methadone maintenance treatment clinics, or remitted patients. We need to be cautious in generalizing findings of the present study due to the limited sample representativeness of Chinese HDPs.

Patient safety is the cornerstone of detoxification treatment for institutionalized HDPs. Health care workers must provide a safe environment for HDPs in China's detoxification institutions. Precautionary measures may include evaluation of the care setting prior to admitting the patient, removal of any items that patients could use to harm themselves, periodical examination of admitted patients' belongings and removing any prohibited items, and reducing patients' chances of accessing prohibited items.

Because of the methodology problems in the available studies, we found a relatively low prevalence of DSH among Chinese HDPs receiving detoxification treatment. Nevertheless, we found that the self-harmers of Chinese HDPs were more likely to harm themselves by using more lethal methods. Given the many negative consequences of DSH and its prevalent methods identified in this meta-analysis, screening for those at risk for DSH and other self-destructive behaviors, strengthening the management of potentially dangerous goods, and maintaining a safe environment should be prioritized for the clinical management of HDPs in China's detoxification treatment settings. Importantly, restricting the availability of DSH methods may be an effective way to prevent or reduce DSH.

## Author contributions

JL: design of the study, interpretation of data for the study, revising the paper critically for important intellectual content, and final approval of the version to be submitted; B-LZ: acquisition and analysis of data for the study, drafting the paper, revising the paper for important intellectual content, and interpretation of data for the study; Y-MX: acquisition and analysis of data for the study, drafting the paper, and interpretation of data for the study; W-XX: analysis of data for the study; W-CC: acquisition and analysis of data for the study, drafting the paper, and revising the paper for important intellectual content.

### Conflict of interest statement

The authors declare that the research was conducted in the absence of any commercial or financial relationships that could be construed as a potential conflict of interest.

## References

[1] Skegg K. Self-harm. Lancet (2005) 366:1471–83. 10.1016/S0140-6736(05)67600-3 16243093

[2] Li YM. Deliberate self-harm and relationship to alcohol use at an emergency department in eastern Taiwan. Kaohsiung J Med Sci. (2007) 23:247–53. 10.1016/S1607-551X(09)70405-X 17525007 PMC11917757

[3] Kimbrel NA Meyer EC DeBeer BB Gulliver SB Morissette SB. The impact of cannabis use disorder on suicidal and nonsuicidal self-injury in iraq/afghanistan-era veterans with and without mental health disorders. Suicide Life Threat Behav. (2018) 48:140–8. 10.1111/sltb.12345 28295524 PMC5597481

[4] Evren C Dalbudak E Evren B Cetin R Durkaya M. Self-mutilative behaviours in male alcohol-dependent inpatients and relationship with posttraumatic stress disorder. Psychiatry Res. (2011) 186:91–6. 10.1016/j.psychres.2010.07.045 20800903

[5] Darke S Torok M Kaye S Ross J. Attempted suicide, self-harm, and violent victimization among regular illicit drug users. Suicide Life Threat Behav. (2010) 40:587–96. 10.1521/suli.2010.40.6.587 21198327

[6] Bergen H Hawton K Waters K Ness J Cooper J Steeg S. Premature death after self-harm: a multicentre cohort study. Lancet (2012) 380:1568–74. 10.1016/S0140-6736(12)61141-6 22995670

[7] Ribeiro JD Franklin JC Fox KR Bentley KH Kleiman EM Chang BP. Self-injurious thoughts and behaviors as risk factors for future suicide ideation, attempts, and death: a meta-analysis of longitudinal studies. Psychol Med. (2016) 46:225–36. 10.1017/S0033291715001804 26370729 PMC4774896

[8] Castellvi P Lucas-Romero E Miranda-Mendizabal A Pares-Badell O Almenara J Alonso I. Longitudinal association between self-injurious thoughts and behaviors and suicidal behavior in adolescents and young adults: a systematic review with meta-analysis. J Affect Disord. (2017) 215:37–48. 10.1016/j.jad.2017.03.035 28315579

[9] Boone SD Brausch AM. Physical activity, exercise motivations, depression, and nonsuicidal self-injury in youth. Suicide Life Threat Behav. (2016) 46:625–33. 10.1111/sltb.12240 26970091

[10] Ronka AR Taanila A Koiranen M Sunnari V Rautio A. Associations of deliberate self-harm with loneliness, self-rated health and life satisfaction in adolescence: Northern Finland Birth Cohort 1986 Study. Int J Circumpolar Health (2013) 72:162–8. 10.3402/ijch.v72i0.21085 23984286 PMC3753134

[11] Ferrey AE Hughes ND Simkin S Locock L Stewart A Kapur N. The impact of self-harm by young people on parents and families: a qualitative study. BMJ Open (2016) 6:e009631. 10.1136/bmjopen-2015-009631 26739734 PMC4716183

[12] United Nations Offic on Drugs and Crime World Drug Report 2017. United Nations publication, Sales No. E.17.XI.6 (2018).

[13] Office of the China National Narcotics Control Commission Illicit Drug Trend in China, 2017 Beijing: CNNCC (2018).

[14] Zhong B Xiang Y Cao X Li Y Zhu J Chiu HF. Prevalence of antisocial personality disorder among Chinese individuals receiving treatment for heroin dependence: a meta-analysis. Shanghai Arch Psychiatry (2014) 26:259–71. 10.11919/j.issn.1002-0829.214091 25477719 PMC4248258

[15] Office of China National Narcotics Control Commission Annual Report on Drug Control in China 2017. Beijing: NNCC (2017).

[16] Saunders KE Hawton K Fortune S Farrell S. Attitudes and knowledge of clinical staff regarding people who self-harm: a systematic review. J Affect Disord. (2012) 139:205–16. 10.1016/j.jad.2011.08.024 21925740

[17] Shaw DG Sandy PT Mental health nurses' attitudes toward self-harm: curricular implications. Health SA Gesondheid (2016) 21:406–14. 10.1016/j.hsag.2016.08.001

[18] Plener PL Allroggen M Kapusta ND Brahler E Fegert JM Groschwitz RC. The prevalence of nonsuicidal self-injury (NSSI) in a representative sample of the German population. BMC Psychiatry (2016) 16:353. 10.1186/s12888-016-1060-x 27760537 PMC5069807

[19] Kerr PL Muehlenkamp JJ Turner JM. Nonsuicidal self-injury: a review of current research for family medicine and primary care physicians. J Am Board Fam Med. (2010) 23:240–59. 10.3122/jabfm.2010.02.090110 20207935

[20] Maloney E Degenhardt L Darke S Nelson EC. Investigating the co-occurrence of self-mutilation and suicide attempts among opioid-dependent individuals. Suicide Life Threat Behav. (2010) 40:50–62. 10.1521/suli.2010.40.1.50 20170261 PMC3073135

[21] Xia S Chi L Zhang X An investigation and analysis of the illicit behavior and its treatment in 1760 cases of heroin abusers during forced withdrawl period. Chin J Drug Abus Prev Treat. (2003) 9:20–1. 10.3969/j.issn.1006-902X.2003.06.007

[22] Shen J Fan N An investigation on reasons of deliberate self-harm in heroin-dependent patients receiving detoxification treatment. J Yunnan Pub Sec Aca (1997) 8:64–5.

[23] Zhu Y Huang F An analysis on the deliberate self-harm behaviors of 32 patients receiving compulsory detoxification. Acta Aca Med Suzhou (1999) 19:936.

[24] Luo S Yang X Li J Preventive strategies of deliberate self-harm of patients under compulsory detoxification. Acta Aca Med Suzhou (2000) 23:87.

[25] Feng X Wang P Causes and prevention of suicide and self-harm in persons under compulsory detoxification. J Zhejiang Pol Coll Pub Sec Sci J. (2002) 7:69–71. 10.3969/j.issn.1674-3040.2002.05.019

[26] Wang J An analysis of deliberate self-harm of 58 patients under compulsory detoxification. Chin J Drug Abus Prev Treat. (2002) 7:28–9. 10.3969/j.issn.1006-902X.2002.02.010

[27] Liu C Causes and preventive strategies for deliberate self-harm of heroin-dependent patients. Chin J Clin Rehab. (2002) 6:3589 10.3321/j.issn:1673-8225.2002.23.096

[28] Yang J The phenomenon of deliberate self-harm of heroin addicts in detention centers. Crim Correc Res. (2003) 3:66–7, 72.

[29] Jia Y An analysis on causing selfinjury of heroin addicts under compulsory detoxification. Chin J Drug Abus Prev Treat. (2003) 9:10–1. 10.3969/j.issn.1006-902X.2003.02.004

[30] Lu Q Zhu Y Causes of self-mutilation of and nursing care for patients under compulsory detoxification. J Nurs Training (2004) 19:380 10.3969/j.issn.1002-6975.2004.04.051

[31] Tao F Wang Q Meng Q Wang H Aggresive behavior of heroin addicts under voluntary detoxification. Shanghai Nurs. (2005) 5:42–3. 10.3969/j.issn.1009-8399.2005.03.021

[32] Zhang Z Zhang Q Deliberate self-harm of opioid-dependent patients and an analysis on 315 cases. Chin Med Res Cin. (2005) 3:86–8.

[33] Wang Y Investigation and analysis of the self-disability behavior in the course of compulsory detoxification. Chin J Drug Abus Prev Treat. (2005) 11:201–3. 10.3969/j.issn.1006-902X.2005.04.005

[34] Wu J Chen T Causes and strategies for deliberate self-harm of 79 cases under compulsory detoxification. Chin J Drug Abus Prev Treat. (2007) 13:106–8. 10.3969/j.issn.1006-902X.2007.02.014

[35] Luo B Self-harm with needles in heroin-dependent patients. Chin J Drug Abus Prev Treat. (2009) 15:342–4. 10.3969/j.issn.1006-902X.2009.06.013

[36] Walker J Holm-Hansen-C, Martin P Sawhney A Thekkumpurath P Beale C. Prevalence of depression in adults with cancer: a systematic review. Ann Oncol. (2013) 24:895–900. 10.1093/annonc/mds575 23175625

[37] Zhong BL Liu TB Chiu HF Chan SS Hu CY Hu XF. Prevalence of psychological symptoms in contemporary Chinese rural-to-urban migrant workers: an exploratory meta-analysis of observational studies using the SCL-90-R. Soc Psychiatry Psychiatr Epidemiol. (2013) 48:1569–81. 10.1007/s00127-013-0672-4 23508367

[38] Cipriano A Cella S Cotrufo P. Nonsuicidal self-injury: a systematic review. Front Psychol. (2017) 8:1946. 10.3389/fpsyg.2017.01946 29167651 PMC5682335

[39] Al-Sharqi AM Sherra KS Al-Habeeb AA Qureshi NA. Suicidal and self-injurious behavior among patients with alcohol and drug abuse. Subst Abuse Rehabil. (2012) 3:91–9. 10.2147/SAR.S22515 24474869 PMC3886647

[40] Verrocchio MC Conti C Fulcheri M. Deliberate self-harm in substance-dependent patients and relationship with alexithymia and personality disorders: a case-control study. J Biol Regul Homeost Agents (2010) 24:461–9. 21122286

[41] Chowdhury S Rahman M Islam M Tabassum R Kamal A Al-Azad M Deliberate self-harm in substance use disorder patients-a study at tertiary level hospitals in Bangladesh. J Armed Forces Med Coll Bangladesh (2013) 9:63–74. 10.3329/jafmc.v9i1.18728

[42] Zhang F Liao H An anaysis of 48 heroin-dependent patients who deliberately self-harm. Zhejiang Clin Med J. (2001) 3:763–4.

[43] Klonsky ED. The functions of self-injury in young adults who cut themselves: clarifying the evidence for affect-regulation. Psychiatry Res. (2009) 166:260–8. 10.1016/j.psychres.2008.02.008 19275962 PMC2723954

[44] Klonsky ED. The functions of deliberate self-injury: a review of the evidence. Clin Psychol Rev. (2007) 27:226–39. 10.1016/j.cpr.2006.08.002 17014942

